# Managing tobacco black shank disease using biochar: direct toxicity and indirect ecological mechanisms

**DOI:** 10.1128/spectrum.00149-24

**Published:** 2024-08-30

**Authors:** Xuan-quan Zhu, Yan Chen, Meng Jia, Hui-juan Dai, Yan-bin Zhou, Huan-wen Yang, Peng Zhou, Yu Du, Ge Wang, Yu-xiang Bai, Na Wang

**Affiliations:** 1 College of Tobacco Science, Yunnan Agricultural University, Kunming, China; 2 China Tobacco Hebei Industrial Co. Ltd., Shijiazhuang, China; Yeungnam University, Gyeongsan, Gyeongbuk, Republic of Korea

**Keywords:** tobacco black shank, biochar, disease suppression, soil microbiome

## Abstract

Black shank disease in tobacco, caused by *Phytophthora nicotianae*, can lead to yield losses of 30%–50% upon outbreak. Recently, biochar derived from agricultural waste has shown significant potential in controlling soil-borne diseases, though its mechanisms remain unclear. Over a 3-year observation period, we found that the incidence of black shank was significantly lower in plots amended with biochar compared with normal cultivation plots. To investigate the underlying mechanisms, we studied both the direct and indirect effects of biochar on black shank. Direct antifungal assays indicated that biochar reduced the total number of sporangia by 53.91%. Further pot experiments revealed a 62.34% reduction in the *P. nicotianae* population in the soil following biochar application. Additionally, biochar application led to notable changes in soil physicochemical properties and microbial community composition. Microbial species analysis showed that biochar promoted the aggregation of beneficial microbes such as *Sphingomonas*, *Flavisolibacter*, and *Mucoromycota*. Functional predictions using the PICRUSt 2 software revealed that biochar enhances bacterial functions related to antimicrobial substance synthesis (Tetracycline biosynthesis), detoxification metabolism (D-arginine and D-ornithine metabolism, arginine and proline metabolism), and lipid and fatty acid metabolism (Lipopolysaccharide biosynthesis, fatty acid biosynthesis), while fungal functions showed no significant changes. This suggests that rhizosphere bacteria play a more prominent role in the suppression of black shank by biochar, a finding supported by partial least squares path modeling analysis. Therefore, we hypothesize that biochar not only directly inhibits *P. nicotianae* growth but also regulates the composition of the rhizosphere microbial community, inducing the production of antimicrobial substances by rhizosphere bacteria, effectively preventing *P. nicotianae* invasion.

**IMPORTANCE:**

Black shank, a global soil-borne fungal disease in tobacco, currently lacks effective control methods. Notably, biochar derived from agricultural waste has shown significant potential in controlling soil-borne diseases. Over a 3-year observation period, we found that plots amended with biochar had a significantly lower incidence of black shank compared with normal cultivation plots. However, the mechanisms of disease suppression remained unclear. Through *in vitro* antifungal assays and pot experiments, we discovered that tobacco-derived biochar can directly inhibit the growth of the pathogen. Additionally, biochar regulates the composition of the rhizosphere microbial community, inducing rhizosphere bacteria to produce antimicrobial substances, effectively preventing pathogen invasion. This discovery reveals both the direct and indirect mechanisms by which biochar suppresses black shank in tobacco. It provides a scientific basis for developing green control technologies for black shank and offers theoretical support for the application of biochar in managing soil-borne diseases in tobacco cultivation areas.

## INTRODUCTION

Tobacco black shank, caused by the soil-borne pathogen *Phytophthora nicotianae*, is a devastating disease globally (1–3), resulting in annual crop losses exceeding a billion dollars (4–6). This pathogen can infect tobacco at both seedling and mature plant stages, leading to stem base and root rot, wilting, and plant death (7). *P. nicotianae* persists in soil or plant debris between crop seasons as thick-walled resistant spores, known as chlamydospores, which can survive for many years in the absence of hosts (3), serving as the primary infection source. Additionally, motile zoospores released from sporangia under warm and wet conditions enable secondary spread within a season. The pathogen also infects other crops, including eggplant, potato, chili, lettuce, olive, and sunflower (8, 9). Despite various control efforts (10–12), effective management strategies against tobacco black shank remain elusive (13).

In China, tobacco black shank has been rapidly spreading, causing 30%–50% or even total yield losses on affected farms (14). Heavy continuous tobacco planting combined with a favorable warm and humid climate in major production areas creates ideal conditions for *P. nicotianae* outbreaks. Moreover, the pathogen has over 50 alternate host crops from the Solanaceae, Cucurbitaceae, and Fabaceae families, aiding its survival and spread (7, 15). Chemical controls have shown limited success against black shank due to the soil-borne nature of the pathogen. Soil fumigants like methyl bromide and chloropicrin are being phased out due to environmental concerns (16–18). While new oomycete-specific chemicals like oxathiapiprolin show promise (19, 20), concerns about fungicide resistance development and adverse ecological impacts remain. Therefore, integrated, sustainable approaches are needed to manage this disease effectively.

Biochar, produced by pyrolyzing biomass waste, enhances soil structure (21–23), water retention (24), nutrient retention and availability (25, 26), soil acidity (27), and microbial communities (28–30). Consequently, it is widely used as a sustainable soil amendment for remediation. Microbes are integral to the disease triangle of hosts, pathogens, and the environment (31), and biochar likely influences this triangle both directly through soil modification and indirectly *via* rhizosphere microbes (32, 33). For instance, biochar can induce systemic resistance, recruit beneficial microbes (34), and suppress soil-borne pathogens such as *Fusarium oxysporum*, *Fusarium proliferatum*, *Ralstonia solanacearum*, and *Aphanomyces euteiches* in various crops (35–39). However, no research has yet explored the prevention and control of black shank disease using biochar.

China annually produces approximately 3–4 million tons of tobacco stem waste from its 1.365 million hectares of tobacco plantations, generating 3 million tons of leaves (40). Currently, this waste is burned, causing pollution. Transforming it into biochar through pyrolysis can provide a sustainable solution within tobacco fields, improving crop yield and quality while suppressing soil pathogens like *P. nicotianae*.

This study evaluated the ability of tobacco stem biochar to inhibit *P. nicotianae* both directly and indirectly through rhizosphere microbes. The objectives were to ascertain direct inhibitory effects, identify shifts in microbial communities, and determine the key microbes involved in black shank suppression.

## MATERIALS AND METHODS

### Investigation of black shank disease

**Fig 1:**
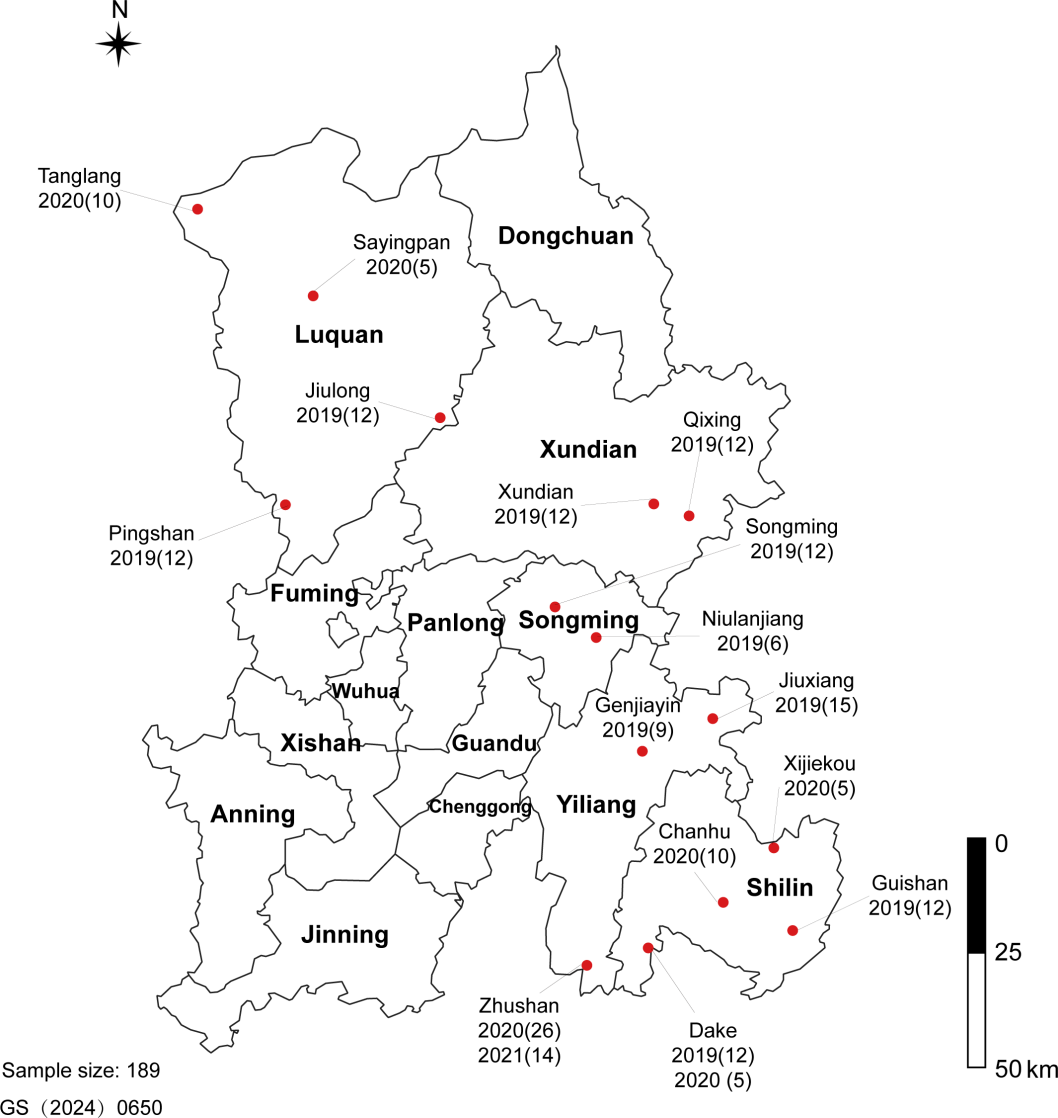
Survey sites for tobacco black shank disease.

From 2019 to 2021, a survey was conducted to investigate the incidence of tobacco black shank disease during its outbreak period in 16 towns across five counties around Kunming, Yunnan Province, China. The disease incidence was calculated as follows: disease incidence (%) = 100 × (diseased plants/total plants). In total, 189 samples were collected, comprising 55 samples treated with conventional compound fertilizer (Control) and 134 samples treated with biochar (Biochar) (Fig. 1). Detailed location information is provided in the attached document.

### Biochar, tobacco seedlings, soil, and *P. nicotianae* preparation

Biochar was produced by the Tobacco Biochemistry Laboratory at Yunnan Agricultural University. Washed tobacco stems were cut into small pieces, naturally dried, and then pyrolyzed at 500°C for 3 hours under oxygen-limited conditions in a muffle furnace (SX2-4-10, Zhonghuan, China). After cooling, the biochar was ground and sieved through a 0.55-mm mesh for later use. The *P. nicotianae* strain, provided by the author’s research group, was purified, propagated, and identified multiple times before use. The tobacco variety used in the experiment was Yunyan 87 (*Nicotiana tabacum*), sourced from the Yunnan Tobacco Research Institute in Yuxi, Yunnan Province, China. Test soil was collected from Azhiying street, Panlong district, Kunming, Yunnan Province (25°24′57″N, 102°49′19″E), from a field with no history of tobacco black shank disease for 2 consecutive years. The soil characteristics included a pH of 5.87, 33.45 g/kg organic matter, 680.67 mg/kg available potassium, 144.04 mg/kg alkaline nitrogen, and 30.95 mg/kg available phosphorus.

### *In vitro* inhibition assay

Potato Dextrose Agar Medium (PDA) preparation involved combining 200 g of potato powder, 20 g of glucose, 15–20 g of agar, and 1,000 mL of distilled water, followed by sterilization at 121°C for 20 minutes.

Biochar Potato Dextrose Agar Medium (BPDA) was prepared by adding 2.5% wt/vol biochar to the PDA medium during its preparation. After autoclaving, the medium was poured into sterile 9-cm-diameter Petri dishes.

For the *in vitro* antibacterial assay, a sterile 5-mm-diameter punch was used to excise the edge of a well-growing activated strain colony, which was then inoculated in the center of the BPDA medium. The PDA medium was inoculated as a control. Both media were incubated at a constant temperature of 30°C for 5 days, with five replicates for each treatment. The number of sporangia was observed and recorded using a microscope (CX23, Olympus, Japan).

### Pot experiments

To prepare *P. nicotianae* suspension, *P. nicotianae* was cultured in PDA at a constant temperature of 30°C until reaching the exponential growth phase. The entire medium and culture were ground and dissolved in sterile water. The mixture was then centrifuged at 3,000 rpm for 10 minutes. The sediment was washed and centrifuged three times to remove the PDA medium, and an appropriate amount of sterile water was added to prepare a bacterial suspension with a concentration of 10^8^ CFU/mL.

Plastic pots with a diameter of 15 cm and a depth of 18 cm were filled with 1,500 g of soil. Biochar was incorporated at a ratio of 2.5% wt/wt, while a control setup excluded biochar. Each treatment involved 30 pots. Seven days after transplanting the tobacco seedlings, 150 mL of the *P. nicotianae* suspension was added to each pot, resulting in a final inoculation level of 10^7^ CFU/g of dry soil. The pots were placed in a greenhouse, and to mitigate localized temperature and humidity variations, the positions of the pots were randomly adjusted every 2 days. Sterile water (200 mL) was added every 5 days to maintain moisture. Thirty days post-inoculation, the tobacco plants were removed, and the rhizosphere soil was collected using the shaking method. The soil from 10 pots was combined to form one replicate, with three replicates per treatment. The soil samples were divided into three parts: one for detecting *P. nicotianae* quantity, another for analyzing soil nutrients and enzyme activity, and the third stored at −80°C for soil microbial analysis.

### DNA extraction, sequencing, and qPCR

Microbial DNA from soil samples was extracted using a HiPure Soil DNA Kit (Magen, Guangzhou, China) according to the manufacturer’s instructions. The 16S rRNA V3-V4 and ITS2 regions from total DNA were amplified using barcoded primers and sequenced on the Illumina platform. Raw reads were filtered for quality, and OTUs were annotated to the SILVA, UNITE, and ITS2 reference databases using an RDP classifier at an 80% confidence level. The raw sequence data reported in this study have been deposited in the Genome Sequence Archive at the National Genomics Data Center, China National Center for Bioinformation/Beijing Institute of Genomics, Chinese Academy of Sciences, under accession project numbers CRA013583 and CRA013584 for 16S rRNA and ITS rRNA genes, respectively, and are publicly accessible at https://bigd.big.ac.cn.

### *P. nicotianae* quantification, soil chemical, and enzymatic analyses

*P. nicotianae* in soil samples was quantified by quantitative real-time polymerase chain reaction (qPCR) detecting system using *P. nicotianae*-specific primers (ITS8-1: 5′-CGAAGCCAACCATACCACGAA-3′, ITS8-2: 5′-ATGAAGAACGCTGCGAACTGC-3′) with three technical replicates and negative controls. The reaction components, thermal cycling, and melt curve conditions followed previously described protocols (41). Soil pH was measured at a 1:2.5 soil-to-water ratio, and soil organic matter, available potassium, available phosphorus, and alkaline nitrogen were estimated according to established methods (42). Soil enzyme activities of catalase, urease, sucrase, and acid phosphatase were determined using standard protocols (43).

### Microbiome network construction

To assess whether biochar addition significantly influenced soil microbial assembly, co-occurrence networks were constructed between OTUs with >1% relative abundance and soil physicochemical parameters using pairwise Spearman correlation analysis in R. Valid links between nodes were defined by correlations with *P* ≤ 0.05 and |*r*| > 0.5 after Benjamini-Hochberg correction. The resulting networks were visualized and analyzed in Gephi 0.9 to compare topological features between treatments.

### Statistical analyses

Maps were created, and sampling locations were marked using QGIS (3.24.1-Tisler), with map data sourced from the National Geographic Information Public Service Platform of the People’s Republic of China, “Tian Di Tu”. All statistical analyses were conducted using R (version 3.4.3). The Shapiro-Wilk test assessed normality, and the *F*-test evaluated the homogeneity of variances before testing for significance. In our analysis of the differences in black shank disease incidence under various management practices in Kunming, we categorized 189 sampling points by year and management practice. The overall incidence of black shank disease for each management practice was represented by the average disease incidence rate of different sampling points each year. We used a *t*-test to assess the significance of these differences. Independent samples *t*-tests were performed to analyze differences in total sporangia count, effective sporangia count, soil chemical properties, and microbial α-diversity between treatments. The top 1,000 species with a relative abundance of ≥0.1% were filtered, and Welch’s *t*-test was employed using the “vegan” package in R to determine genus-level microbial differences. Non-metric multi-dimensional scaling (NMDS) analysis based on Bray-Curtis dissimilarity was performed using the “vegan” package to analyze microbial community differences under various treatments. Bacterial function prediction was annotated using the KEGG Pathway in PICRUSt2 software, and fungal function was annotated using the MetaCyc Pathway. Welch’s *t*-test was again utilized *via* the “vegan” package to determine differential functions of bacteria and fungi. Partial least squares path modeling (PLS-PM) using the “plspm” package in R evaluated the relationships among biochar, soil properties, microbiome, *P. nicotianae*, and disease incidence. Path coefficients and *R*² values were estimated and validated using bootstrap methods (10,000 iterations), excluding latent variables with loading values < 0.7 to ensure model usability. Finally, the overall predictive performance of the model was evaluated using Goodness of Fit (GOF) indicators.

## RESULTS

### Disease investigation in field condition

**Fig 2:**
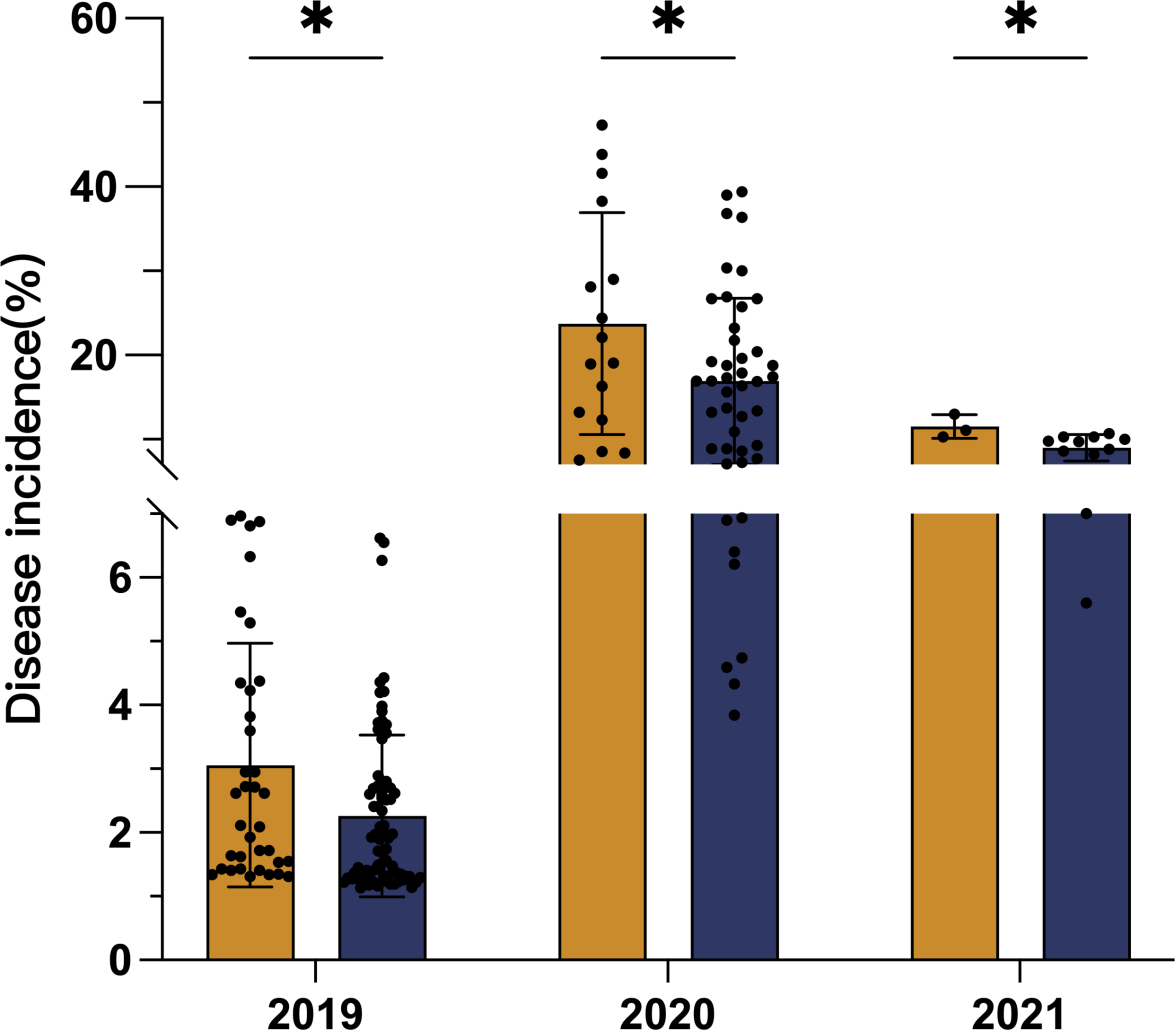
Effect of biochar application on the incidence of tobacco black shank disease in Kunming tobacco-growing areas from 2019 to 2021. We averaged the incidence rates from different sampling points each year to represent the overall incidence of black shank disease under different management practices. The dots within the bars indicate the incidence rate at each sampling point. The error bars represent the standard error of the mean. * indicates a significant difference at the 0.05 level.

A survey was conducted in 189 tobacco fields in Kunming, Yunnan Province, China (Fig. 1), located between 24°34′−26°11′N latitude and 102°17′−103°33′E longitude, at an altitude of 1,492–2,250 meters. The cultivated varieties included Honghuadajinyuan, K326, Yun87, and NC102 (Table S1). Black shank disease was observed annually from 2019 to 2021, with the highest incidence recorded in 2020 and a relatively lower incidence in 2019. Notably, a consistent trend of significantly lower disease incidence was observed in fields treated with biochar compared with untreated fields (Fig. 2, *P* < 0.05), with a reduction range of 21.53%–28.63%. To elucidate this phenomenon, the underlying mechanisms by which biochar controls black shank disease were investigated through direct antibacterial assays and pot experiments.

### Effect of biochar on the growth of *P. nicotianae*

**Fig 3:**
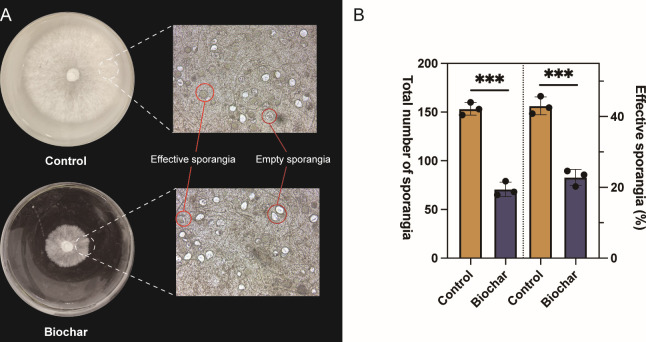
Effect of different treatments on the growth of *P. nicotianae* during *in vitro* assay. (**A**) Growth of tobacco phytophthora: the left image shows the growth of *P. nicotianae* in the medium after 5 days and the right image shows *P. nicotianae* sporangia under an optical microscope. (**B**) Sporangia and effective sporangium count in control and biochar treatments. The left y-axis represents the total number of sporangia, while the right y-axis represents the percentage of effective sporangia. The error bars represent the standard error of the mean. ***, **, and * indicate significant differences at the 0.001 level, 0.01 level, and 0.05 level, respectively.

To comprehend the potential effects of biochar on *P. nicotianae*, its impact on the pathogen’s growth and sporulation was investigated. *In vitro* assays demonstrated that incorporating biochar significantly inhibited *P. nicotianae* growth and sporulation (Fig. 3A). Additionally, both the total sporangia count and the proportion of effective sporangia decreased compared with those of the control (Fig. 3B). These findings indicate that biochar exerts a strong antifungal effect against *P. nicotianae*, reducing its growth and ability to produce infectious spores.

### Indirect effects of biochar on black shank disease incidence

#### Biochar reduces *P. nicotianae* colonization and alters soil properties

**Fig 4:**
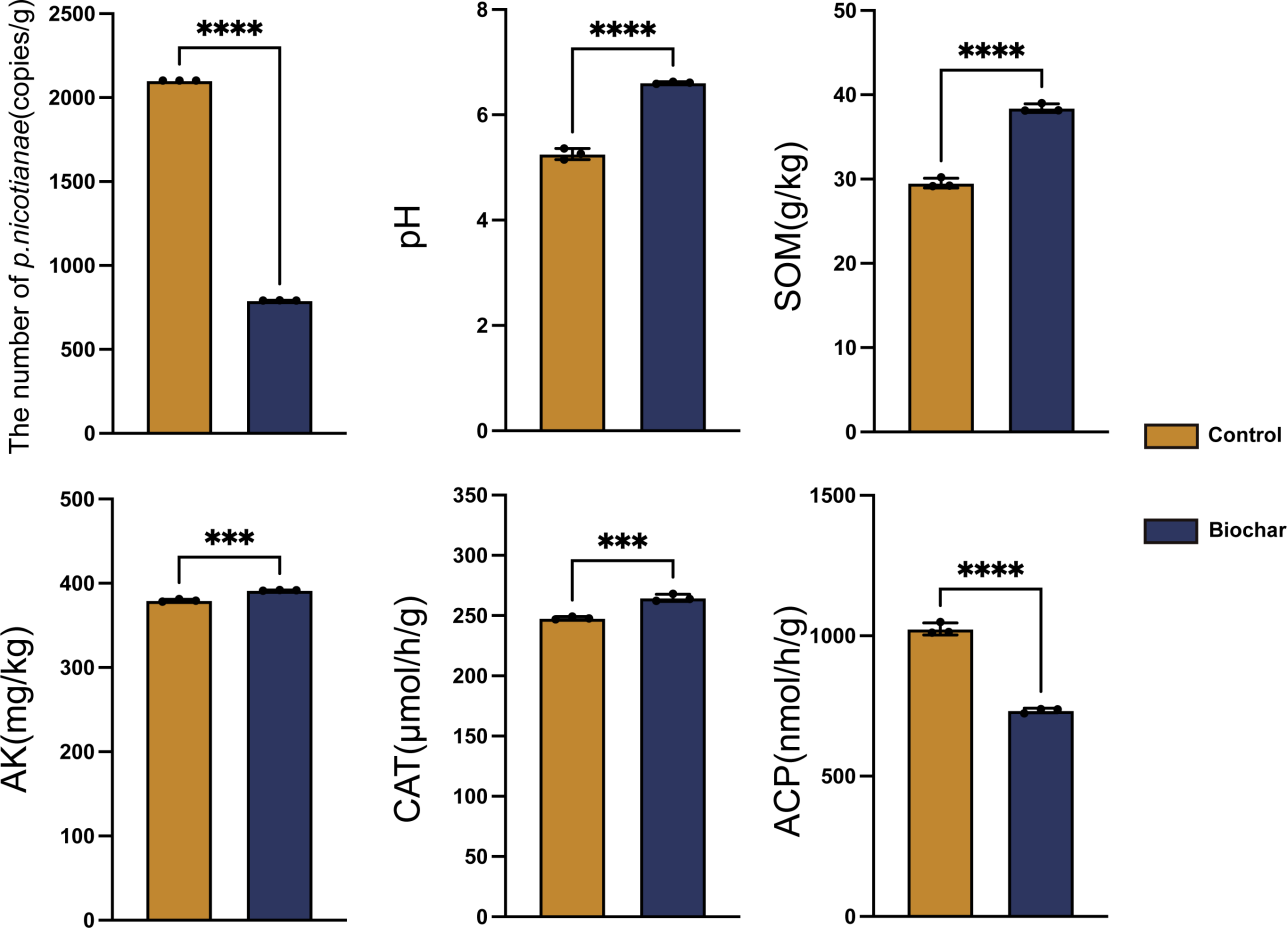
Effect of biochar application on soil *P. nicotianae* quantity and chemical properties. SOM represents soil organic matter content, AK represents available potassium content, CAT represents catalase activity, and ACP represents acid phosphatase activity.

Quantification of *P. nicotianae* by qPCR revealed that biochar amendment significantly reduced the number of pathogens in rhizosphere soil compared with unamended controls. The average *P. nicotianae* copy numbers decreased by 62.34% in rhizosphere soil with biochar treatment (Fig. 4).

Additionally, incorporating biochar improved several soil physicochemical properties (Fig. 4). Compared with the control, biochar significantly increased soil pH, soil organic matter (SOM), and available potassium (AK) in rhizosphere soil. SOM content showed a substantial increment of 30.14%, while AK levels rose by 3.18%. Regarding enzyme activities, catalase (CAT) activity significantly increased, whereas acid phosphatase (ACP) activity decreased in rhizosphere soil amended with biochar (Fig. 4).

#### Effects of biochar application on soil microbial community structure and function

**Fig 5:**
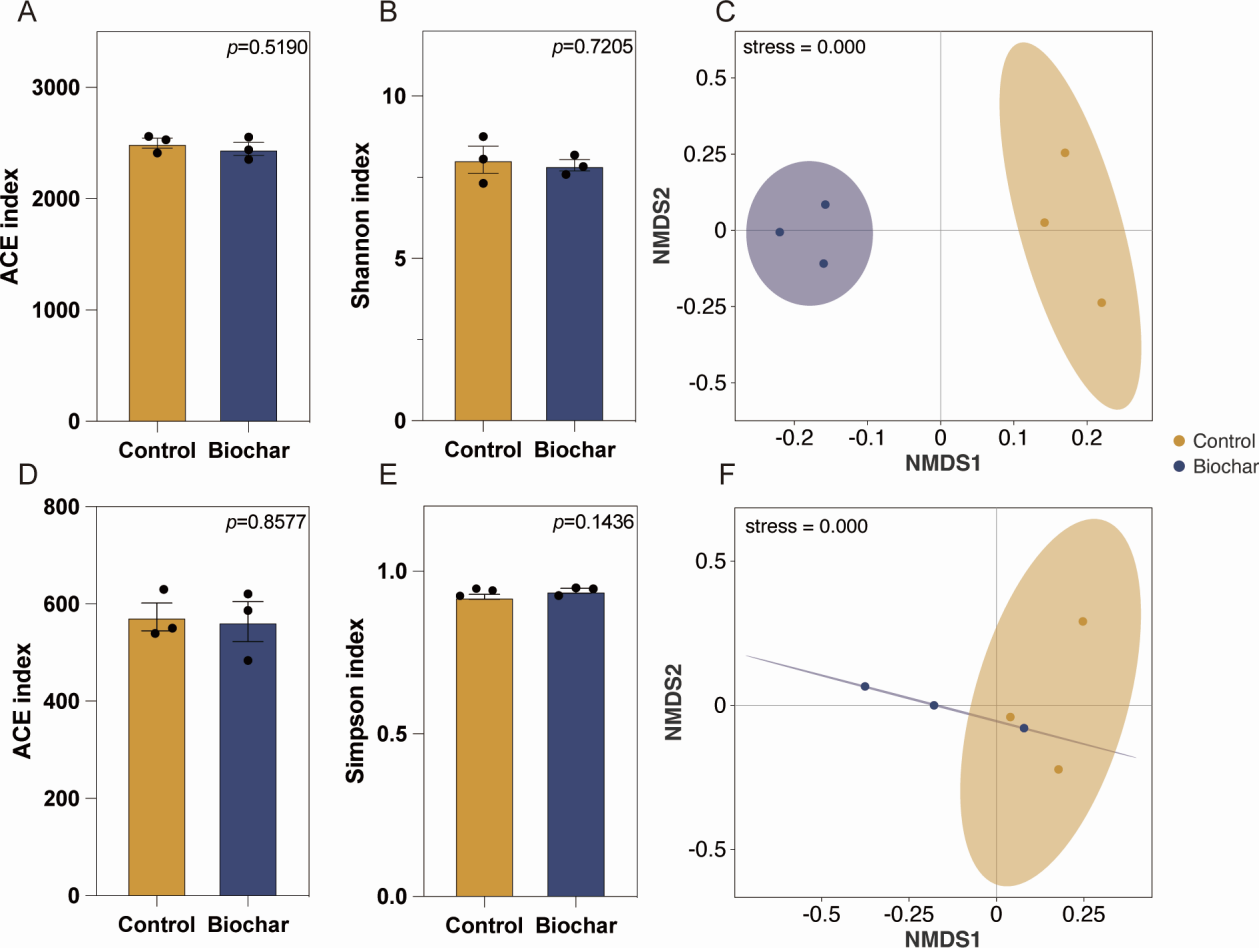
α and β diversity analyses for bacterial and fungal communities. (**A** and **B**) Effects of biochar application on soil bacterial community diversity. Abundance-based coverage estimator (ACE) and Shannon indices represent soil microbial diversity, with a higher ACE index indicating greater species richness and a higher Shannon index indicating greater diversity. (**C**) Effect of biochar application on soil bacterial community composition. NMDS represents NMDS. (**D** and **E**) Effects of biochar application on soil fungal community diversity. (**F**) Effect of biochar application on soil fungal community composition.

A total of 81,457 raw sequences were obtained from all samples, forming 2,203 OTUs at the 97% similarity threshold. Biochar application led to a reduction in the α-diversity of soil bacteria and fungi (Fig. 5A, B, D and E), though the decrease was not statistically significant. Microbial β-diversity analysis revealed substantial shifts in community composition following Bioahr application (Fig. 5C and F). NMDS plots indicated that bacterial communities separated into two distinct clusters (Fig. 5C, stress < 0.001), while fungal communities exhibited closer clustering with less distinction between treatments (Fig. 5F).

**Fig 6:**
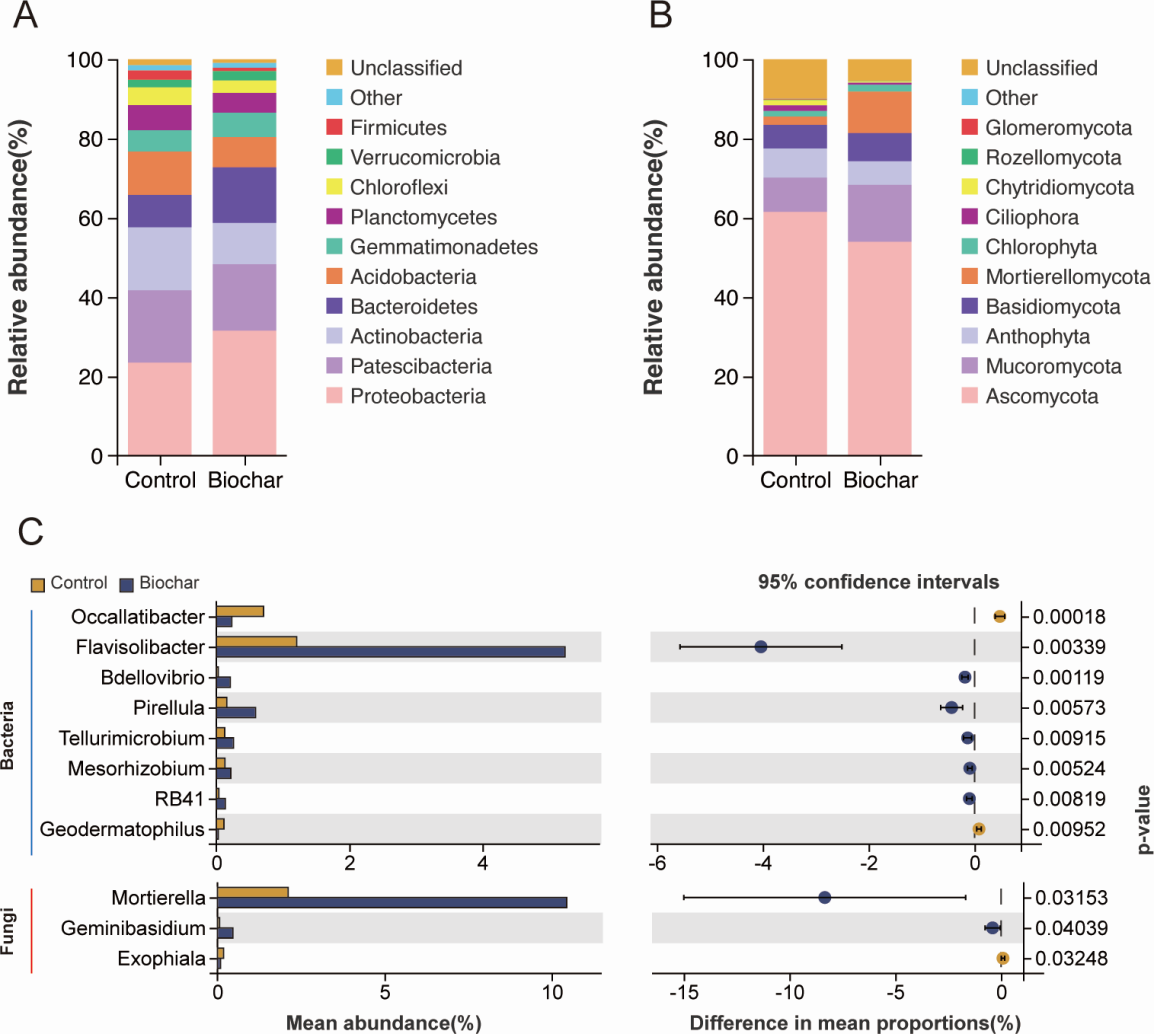
Effects of biochar application on soil microbial composition. (**A**) Top 10 bacterial phyla composition. (**B**) Top 10 fungal phyla composition. (**C**) Differential analysis of species at the genus level. The left part of the graph displays differential species on the y-axis and mean abundance on the x-axis; the right part shows the abundance difference between groups on the x-axis, with dot color indicating the group with higher abundance and error bars representing the 95% CI of the difference. Blue lines indicate bacteria, and red lines indicate fungi.

The bacterial community in biochar-treated soils predominantly comprised Proteobacteria, Patescibacteria, Actinobacteria, Bacteroidetes, Acidobacteria, and Gemmatimonadetes (Fig. 6A). At the genus level, differential analysis showed that biochar application significantly decreased the abundance of *Occallatibacter* and *Geodermatophilus*, while significantly increasing the abundance of *Flavisolibacter*, *Bdellovibrio*, *Pirellula*, *Tellurimicrobium*, *Mesorhizobium*, and *RB41* (Fig. 6C). The fungal community mainly consisted of Ascomycota, *Mucoromycota*, Anthophyta, Basidiomycota, and Mortierellomycota (Fig. 6B). Biochar treatment significantly reduced the relative abundance of *Mortierella* and *Geminibasidium*, while increasing the relative abundance of *Exophiala*. These findings suggest that biochar significantly impacts microbial diversity and community composition under pathogen stress (Fig. 6C).

**Fig 7:**
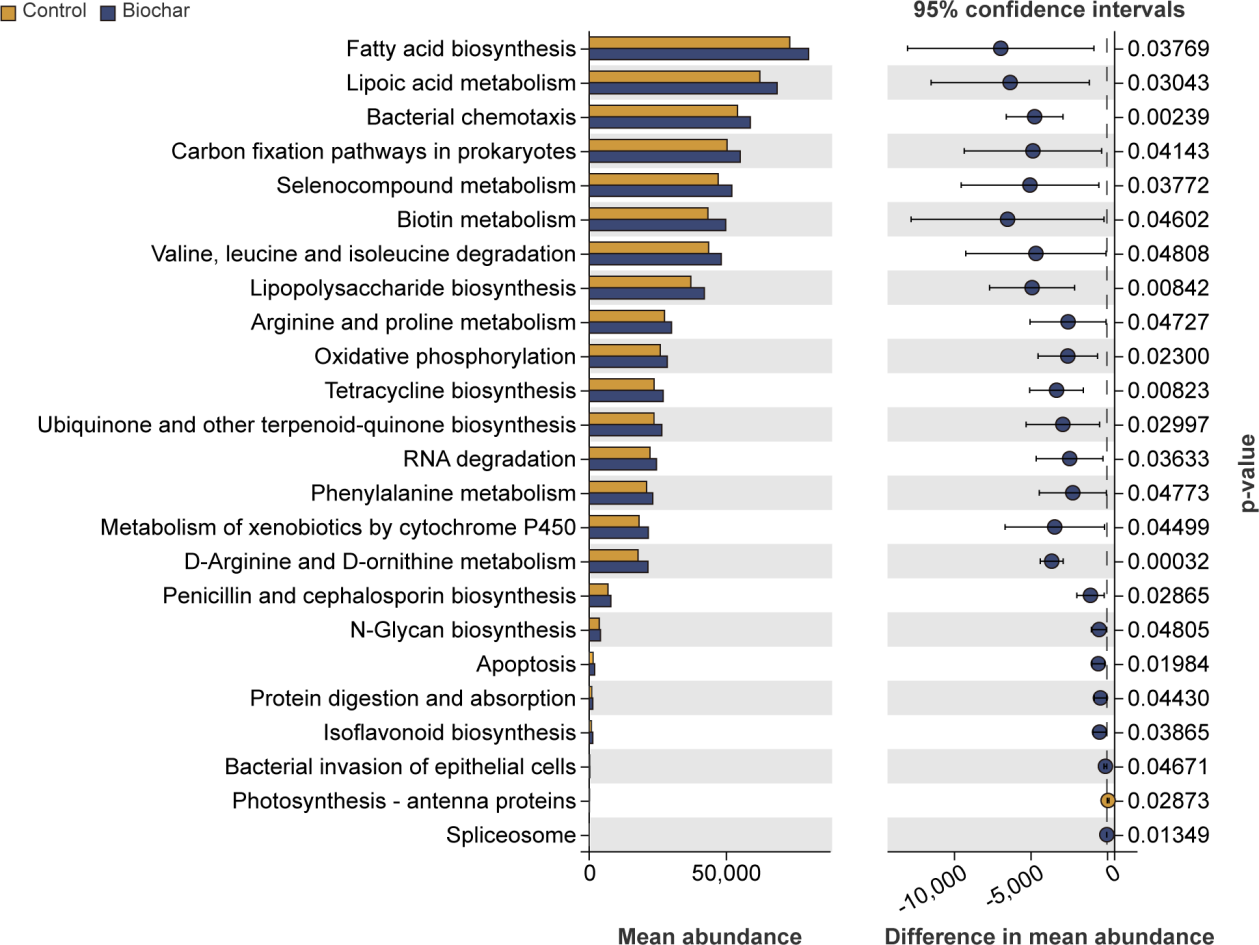
Effects of biochar application on soil bacterial functions. The left part of the graph displays significantly different bacterial functions (*P* < 0.05) on the y-axis and the abundance of these differential functions on the x-axis. The right part displays the confidence interval range of functional abundance differences between groups on the x-axis, with color indicating the group with higher abundance and the y-axis representing the *P* value.

To understand the functional differences in microbial communities under different treatments, PICRUSt 2 was used to predict the potential metabolic capabilities of soil microbes. The results indicated that biochar application significantly affected soil bacterial functions, notably increasing the abundance of functions related to the synthesis of antibacterial substances (tetracycline biosynthesis), the metabolism of detoxifying substances (D-arginine and D-ornithine metabolism, arginine and proline metabolism), and lipid and fatty acid metabolism (lipopolysaccharide biosynthesis, fatty acid biosynthesis). Conversely, the abundance of functions related to photosynthesis-antenna proteins significantly decreased (Fig. 7). No significant effects on fungal functions were observed (Table S2).

### Microbiome networking analysis

**Fig 8:**
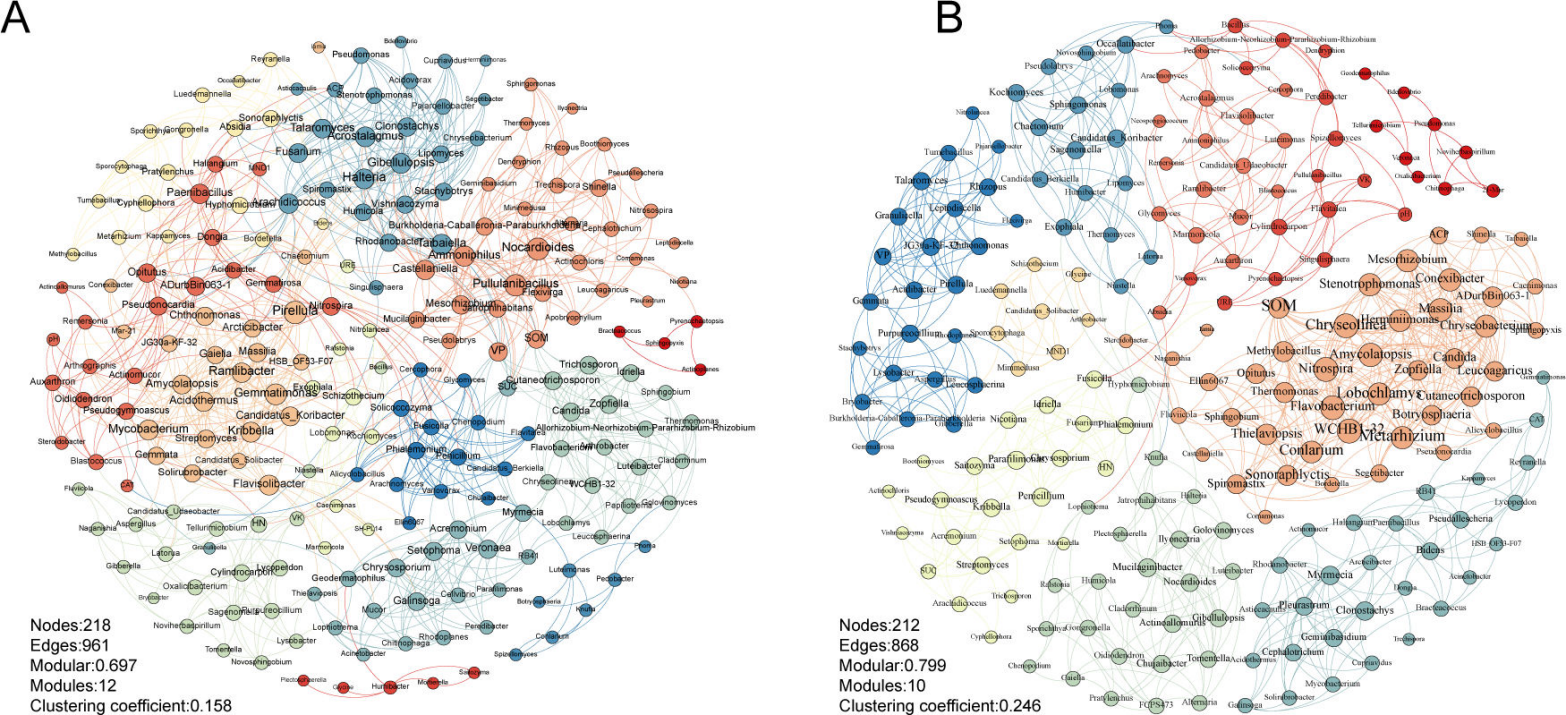
Soil microbial co-occurrence network analysis. (**A**) Microbial co-occurrence network in the control treatment. (**B**) Microbial co-occurrence network in the biochar treatment. Nodes and edges are colored by modules, with node size proportional to degree and edge thickness proportional to Spearman correlation coefficients.

In the rhizosphere network (Fig. 8A and B), the addition of biochar decreased the number of nodes, edges, and modules, while increasing the modularity and clustering of network nodes, indicating greater connectivity. The top 10 most influential species varied between treatments, with more beneficial bacteria and saprophytic fungi present in the biochar treatment. Overall, biochar integration enhanced network stability by altering the topological structure and reconfiguring interactions between microbial communities in both soils.

### PLS-PM analysis

**Fig 9:**
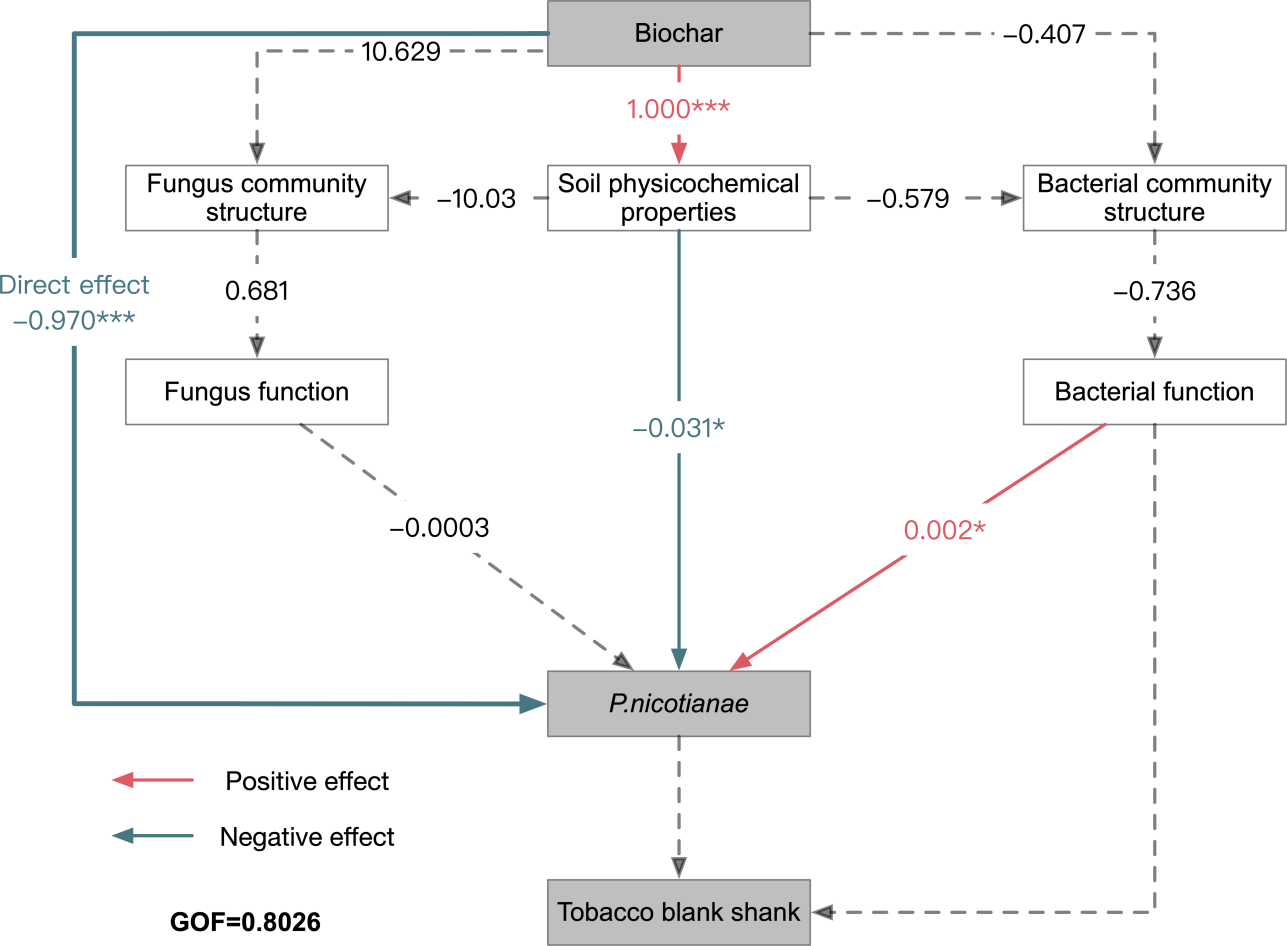
PLS-PM analysis of the relationships among biochar, soil properties, microbiome, *P. nicotianae*, and disease incidence. Biochar indicates biochar application status, with “1” for application and “0” for no application. Soil properties include CAT, ACP, pH, SOM, HN, VP, and VK. Fungal community composition is represented by NMDS3, and fungal functions include the top 20 functions annotated by PICRUSt 2. Bacterial community composition is represented by NMDS1, and bacterial functions include the top 20 functions annotated by PICRUSt 2. *P. nicotianae* represents the copy number of *P. nicotianae* in the soil. Path coefficients were estimated with 10,000 bootstraps. Positive and negative effects are indicated by orange and green arrows, respectively. Non-significant path coefficients are shown as gray dashed lines; *P < 0.05, **P < 0.01, and ***P < 0.001. The model was evaluated using the GOF value.

PLS-PM analysis elucidated the relationships between biochar, soil properties, microbiome, *P. nicotianae*, and disease incidence (Fig. 9). The GOF score was 0.8026, indicating the reliability of the constructed structural equation model. Biochar exhibited a direct negative effect on *P. nicotianae* (path = −0.979, *P* < 0.01) and a direct positive effect on soil physicochemical properties (path = −1.000, *P* < 0.01). Additionally, biochar positively influenced the fungal community structure and negatively influenced the bacterial community structure, though these effects were not significant. Bacterial function significantly impacted *P. nicotianae* positively (path = 0.002, *P* < 0.01), whereas fungal function showed no significant effect on *P. nicotianae*. These results suggest that bacterial function plays a crucial role in inhibiting *P. nicotianae* proliferation, while fungal community function is not significantly associated with disease incidence.

## DISCUSSION

The research and application of biochar in soil remediation have garnered widespread attention (44–47). However, the complexity of soil environments limits our understanding of the mechanisms by which biochar functions, hindering its efficient utilization (48). This study surveyed 189 tobacco fields in Kunming, Yunnan Province, from 2019 to 2021, revealing a significant 25.08% reduction in tobacco black shank disease incidence following biochar application. To elucidate the effects of biochar on disease incidence, this research systematically explored the micro-ecological mechanisms through both direct and indirect actions, a perspective not extensively covered in previous studies. The findings provide new insights into using biochar to mitigate soil-borne pathogens and offer a theoretical basis for the scientific utilization of tobacco stem waste in tobacco-growing regions.

Indoor antibacterial tests visually confirmed that biochar can directly affect pathogens. The growth and sporangia counts of *P. nicotianae* were significantly reduced in the BPDA (Biochar Potato Dextrose Agar) medium, indicating that biochar addition can directly inhibit *P. nicotianae* growth. This inhibition may be related to the inherent properties of biochar, which, due to its porous structure, adsorbs *P. nicotianae* on its surface, limiting its proliferation (49–51). Additionally, biochar’s surface contains numerous functional groups and partially decomposed organic substances that may exert direct toxicity on *P. nicotianae* (13, 52, 53). Although these results demonstrate that biochar can reduce *P. nicotianae* activity under laboratory conditions, they do not fully explain the underlying mechanisms of biochar’s inhibitory effects on black shank disease. Therefore, pot experiments were conducted under controlled conditions, focusing on soil environment factors while controlling for the host and pathogen.

The pot experiment results demonstrated that biochar application significantly reduced the quantity of *P. nicotianae* in the soil environment. However, the mechanisms underlying disease suppression in soil are evidently more complex than the direct effects alone (54, 55). Biochar has been reported to enhance the activity and composition of saprophytic soil microorganisms by improving abiotic factors, such as organic matter and pH, thereby suppressing disease occurrence (56). It can also induce changes in enzyme activity, affecting soil element cycling associated with microorganisms and enhancing soil (57) or plant disease resistance (58). These studies suggest that changes in the soil micro-ecological environment induced by biochar, particularly alterations in microbial community structure and function, may have direct and indirect causal relationships with disease incidence. Based on this, the impact of biochar on the soil micro-ecological environment was further explored.

Rhizosphere microbes play a crucial role in maintaining soil health and form the first line of defense against soil-borne pathogens (59). The study found that biochar increased the relative abundance of bacterial genera such as *Sphingomonas* and *Flavisolibacter*, as well as fungal genera such as *Mortierella*, *Mucoromycota*, Basidiomycota, and *Mortierellomycota*. Microbial differential analysis confirmed that biochar significantly altered the relative abundance of *Flavisolibacter* and *Mortierella*, suggesting that these microbes may play important roles in the process by which biochar inhibits pathogen infection. The presence of *Sphingomonas*, *Flavisolibacter*, and *Mortierella* has also been observed in many healthy soils, where these microbes can reduce disease incidence by enhancing plant disease resistance, occupying pathogen invasion sites, or producing antibacterial substances (60–63). Therefore, it is hypothesized that under pathogen stress, biochar promotes the recruitment of beneficial bacteria to the rhizosphere, effectively preventing pathogen invasion by enhancing plant resistance or inhibiting pathogen activity. Functional prediction confirmed this hypothesis, showing significant increases in the abundance of functions related to antibacterial substance synthesis (tetracycline biosynthesis), detoxification metabolism (D-arginine and D-ornithine metabolism, arginine and proline metabolism), and lipid and fatty acid metabolism (lipopolysaccharide biosynthesis, fatty acid biosynthesis) in rhizosphere soil after biochar application. Substances such as lipopolysaccharides and fatty acids are involved in the construction of cell membranes (64, 65). Evidence suggests that black shank disease pathogens need to alter or disrupt cell membrane permeability to invade roots *via* hyphae or sporangia (66). Additionally, material exchange and conversion between rhizosphere soil microbes and plants must occur (67, 68). Therefore, it is speculated that the beneficial bacteria recruited by biochar may help strengthen root cell membranes by accelerating the synthesis of lipopolysaccharides and fatty acids, thereby preventing pathogen infection.

Microbes in soil do not exist in isolation but are interconnected through various direct and indirect ecological processes such as cooperation, competition, and antagonism, forming complex microbial symbiotic networks (69). Comparing microbial symbiotic networks under different soil conditions revealed that biochar application decreased the number of nodes, edges, and modules while increasing the modularity coefficient and clustering degree of the network. This change may be related to the enrichment of the tetracycline biosynthesis function, as tetracycline is a broad-spectrum antibiotic (70) that inhibits the growth of various species, including pathogens, leading to a reduction in nodes and edges after biochar application. Furthermore, many studies have shown that the loss of network nodes and edges reduces network complexity but accelerates information exchange between nodes (71, 72). Therefore, it is hypothesized that under black shank disease infection conditions, biochar application disrupts the original microbial network but reconstructs a new network capable of rapidly responding to subsequent pathogen infections. Unfortunately, current results cannot fully substantiate this hypothesis. Our research team is currently investigating the response mechanisms of soil microbial communities under continuous pathogen infection conditions to address unresolved issues from this study.

Biochar effectively inhibits the pathogen responsible for tobacco black shank disease by suppressing its growth and reducing sporangia viability. Additionally, biochar enhances the recruitment of beneficial bacteria such as *Sphingomonas*, *Flavisolibacter*, and *Mucoromycota* to the rhizosphere. This recruitment process prevents pathogen invasion by boosting the synthesis of antibacterial substances (tetracycline biosynthesis), detoxification metabolism (D-arginine and D-ornithine metabolism, arginine and proline metabolism), and lipid and fatty acid metabolism (lipopolysaccharide biosynthesis, fatty acid biosynthesis) in rhizosphere soil.

## SUPPLEMENTAL MATERIAL

Supplemental material spectrum.00149-24-s0001.docx Tables S1 and S2.

ASM does not own the copyrights to Supplemental Material that may be linked to, or accessed through, an article. The authors have granted ASM a non-exclusive, world-wide license to publish the Supplemental Material files. Please contact the corresponding author directly for reuse.
