# Supplementary material for: Managing tobacco black shank disease using biochar: direct toxicity and indirect ecological mechanisms
Source: Microbiol Spectr. 2024 Aug 30;12(10):e00149-24. doi: 10.1128/spectrum.00149-24 (PMC11448098; doi:10.1128/spectrum.00149-24)
Supplement: Supplemental material — Tables S1 and S2. [file spectrum.00149-24-s0001.docx]

**Table S1. Information on Survey Sites for Black Shank Disease Incidence**

| Year | Province | City | County | Villages | Lon | Lat | Elevation(m) | Cultivar | Biochar strategy | Incidence of black shank disease(%) |
| --- | --- | --- | --- | --- | --- | --- | --- | --- | --- | --- |
| 2019 | Yunnan | Kunming | Luquan | Jiulong | 102.81 | 25.75 | 2020 | Yunyan87 | control | 1.31 |
| 2019 | Yunnan | Kunming | Luquan | Jiulong | 102.81 | 25.75 | 2020 | Yunyan87 | control | 1.34 |
| 2019 | Yunnan | Kunming | Luquan | Jiulong | 102.81 | 25.75 | 2020 | Yunyan87 | control | 1.40 |
| 2019 | Yunnan | Kunming | Luquan | Pingshan | 102.47 | 25.56 | 1720 | Yunyan87 | control | 1.31 |
| 2019 | Yunnan | Kunming | Luquan | Pingshan | 102.47 | 25.56 | 1720 | Yunyan87 | control | 1.34 |
| 2019 | Yunnan | Kunming | Luquan | Pingshan | 102.47 | 25.56 | 1720 | Yunyan87 | control | 1.40 |
| 2020 | Yunnan | Kunming | Luquan | Sayingpan | 102.53 | 26.01 | 2250 | Honghuadajinyuan | control | 47.31 |
| 2020 | Yunnan | Kunming | Luquan | Sayingpan | 102.53 | 26.01 | 2250 | Honghuadajinyuan | control | 43.85 |
| 2020 | Yunnan | Kunming | Luquan | Tanglang | 102.29 | 26.19 | 1970 | Honghuadajinyuan | control | 22.11 |
| 2020 | Yunnan | Kunming | Luquan | Tanglang | 102.29 | 26.19 | 1970 | Honghuadajinyuan | control | 19.03 |
| 2020 | Yunnan | Kunming | Luquan | Tanglang | 102.29 | 26.19 | 1970 | Yunyan87 | control | 8.40 |
| 2020 | Yunnan | Kunming | Luquan | Tanglang | 102.29 | 26.19 | 1970 | Yunyan87 | control | 7.55 |
| 2020 | Yunnan | Kunming | Shilin | Dake | 103.25 | 24.61 | 1643.81 | Honghuadajinyuan | control | 38.26 |
| 2020 | Yunnan | Kunming | Shilin | Dake | 103.25 | 24.61 | 1643.81 | Honghuadajinyuan | control | 28.13 |
| 2019 | Yunnan | Kunming | Shilin | Dake | 103.25 | 24.61 | 1643.81 | K326 | control | 1.64 |
| 2019 | Yunnan | Kunming | Shilin | Dake | 103.25 | 24.61 | 1643.81 | K326 | control | 1.55 |
| 2019 | Yunnan | Kunming | Shilin | Dake | 103.25 | 24.61 | 1643.81 | K326 | control | 1.93 |
| 2019 | Yunnan | Kunming | Shilin | Guishan | 103.56 | 24.65 | 1850 | K326 | control | 1.35 |
| 2019 | Yunnan | Kunming | Shilin | Guishan | 103.56 | 24.65 | 1850 | K326 | control | 1.43 |
| 2019 | Yunnan | Kunming | Shilin | Guishan | 103.56 | 24.65 | 1850 | K326 | control | 1.72 |
| 2020 | Yunnan | Kunming | Shilin | Xijiekou | 103.52 | 24.83 | 1997.2 | Honghuadajinyuan | control | 28.98 |
| 2020 | Yunnan | Kunming | Shilin | Xijiekou | 103.52 | 24.83 | 1997.2 | Honghuadajinyuan | control | 24.38 |
| 2020 | Yunnan | Kunming | Shilin | Chanhu | 103.41 | 24.71 | 1838.67 | NC102 | control | 16.32 |
| 2020 | Yunnan | Kunming | Shilin | Chanhu | 103.41 | 24.71 | 1838.67 | NC102 | control | 8.56 |
| 2020 | Yunnan | Kunming | Shilin | Chanhu | 103.41 | 24.71 | 1838.67 | NC102 | control | 18.96 |
| 2020 | Yunnan | Kunming | Shilin | Chanhu | 103.41 | 24.71 | 1838.67 | NC102 | control | 12.33 |
| 2019 | Yunnan | Kunming | Songming | - | 103.05 | 25.34 | 2100 | K326 | control | 2.72 |
| 2019 | Yunnan | Kunming | Songming | - | 103.05 | 25.34 | 2100 | K326 | control | 2.62 |
| 2019 | Yunnan | Kunming | Songming | - | 103.05 | 25.34 | 2100 | K326 | control | 2.95 |
| 2019 | Yunnan | Kunming | Songming | Niulanjiang | 103.14 | 25.28 | 1850 | K326 | control | 6.33 |
| 2019 | Yunnan | Kunming | Songming | Niulanjiang | 103.14 | 25.28 | 1850 | K326 | control | 6.81 |
| 2019 | Yunnan | Kunming | Songming | Niulanjiang | 103.14 | 25.28 | 1850 | K326 | control | 6.97 |
| 2019 | Yunnan | Kunming | Xundian | - | 103.26 | 25.56 | 1762 | Yunyan87 | control | 3.82 |
| 2019 | Yunnan | Kunming | Xundian | - | 103.26 | 25.56 | 1762 | Yunyan87 | control | 3.60 |
| 2019 | Yunnan | Kunming | Xundian | - | 103.26 | 25.56 | 1762 | Yunyan87 | control | 4.35 |
| 2019 | Yunnan | Kunming | Xundian | Qixing | 103.34 | 25.54 | 1990 | Yunyan87 | control | 2.62 |
| 2019 | Yunnan | Kunming | Xundian | Qixing | 103.34 | 25.54 | 1990 | Yunyan87 | control | 2.72 |
| 2019 | Yunnan | Kunming | Xundian | Qixing | 103.34 | 25.54 | 1990 | Yunyan87 | control | 2.95 |
| 2019 | Yunnan | Kunming | Yiliang | Genjiayin | 103.24 | 25.03 | 1492 | K326 | control | 4.37 |
| 2019 | Yunnan | Kunming | Yiliang | Genjiayin | 103.24 | 25.03 | 1492 | K326 | control | 4.22 |
| 2019 | Yunnan | Kunming | Yiliang | Genjiayin | 103.24 | 25.03 | 1492 | K326 | control | 5.46 |
| 2019 | Yunnan | Kunming | Yiliang | Genjiayin | 103.24 | 25.03 | 1492 | K326 | control | 5.29 |
| 2019 | Yunnan | Kunming | Yiliang | Genjiayin | 103.24 | 25.03 | 1492 | K326 | control | 6.90 |
| 2019 | Yunnan | Kunming | Yiliang | Genjiayin | 103.24 | 25.03 | 1492 | K326 | control | 6.88 |
| 2019 | Yunnan | Kunming | Yiliang | Jiuxiang | 103.39 | 25.10 | 1920 | K326 | control | 1.72 |
| 2019 | Yunnan | Kunming | Yiliang | Jiuxiang | 103.39 | 25.10 | 1920 | K326 | control | 1.53 |
| 2019 | Yunnan | Kunming | Yiliang | Jiuxiang | 103.39 | 25.10 | 1920 | K326 | control | 1.62 |
| 2019 | Yunnan | Kunming | Yiliang | Jiuxiang | 103.39 | 25.10 | 1920 | K326 | control | 1.43 |
| 2019 | Yunnan | Kunming | Yiliang | Jiuxiang | 103.39 | 25.10 | 1920 | K326 | control | 2.11 |
| 2019 | Yunnan | Kunming | Yiliang | Jiuxiang | 103.39 | 25.10 | 1920 | K326 | control | 2.09 |
| 2020 | Yunnan | Kunming | Yiliang | Zhushan | 103.12 | 24.58 | 1650 | Honghuadajinyuan | control | 13.20 |
| 2020 | Yunnan | Kunming | Yiliang | Zhushan | 103.12 | 24.58 | 1650 | Honghuadajinyuan | control | 41.57 |
| 2021 | Yunnan | Kunming | Yiliang | Zhushan | 103.12 | 24.58 | 1650 | Honghuadajinyuan | control | 13.00 |
| 2021 | Yunnan | Kunming | Yiliang | Zhushan | 103.12 | 24.58 | 1650 | Honghuadajinyuan | control | 11.10 |
| 2021 | Yunnan | Kunming | Yiliang | Zhushan | 103.12 | 24.58 | 1650 | Honghuadajinyuan | control | 10.30 |
| 2019 | Yunnan | Kunming | Luquan | Jiulong | 102.81 | 25.75 | 2020 | Yunyan87 | Biochar | 1.26 |
| 2019 | Yunnan | Kunming | Luquan | Jiulong | 102.81 | 25.75 | 2020 | Yunyan87 | Biochar | 1.19 |
| 2019 | Yunnan | Kunming | Luquan | Jiulong | 102.81 | 25.75 | 2020 | Yunyan87 | Biochar | 1.16 |
| 2019 | Yunnan | Kunming | Luquan | Jiulong | 102.81 | 25.75 | 2020 | Yunyan87 | Biochar | 1.29 |
| 2019 | Yunnan | Kunming | Luquan | Jiulong | 102.81 | 25.75 | 2020 | Yunyan87 | Biochar | 1.22 |
| 2019 | Yunnan | Kunming | Luquan | Jiulong | 102.81 | 25.75 | 2020 | Yunyan87 | Biochar | 1.23 |
| 2019 | Yunnan | Kunming | Luquan | Jiulong | 102.81 | 25.75 | 2020 | Yunyan87 | Biochar | 1.35 |
| 2019 | Yunnan | Kunming | Luquan | Jiulong | 102.81 | 25.75 | 2020 | Yunyan87 | Biochar | 1.28 |
| 2019 | Yunnan | Kunming | Luquan | Jiulong | 102.81 | 25.75 | 2020 | Yunyan87 | Biochar | 1.30 |
| 2019 | Yunnan | Kunming | Luquan | Pingshan | 102.47 | 25.56 | 1720 | Yunyan87 | Biochar | 1.26 |
| 2019 | Yunnan | Kunming | Luquan | Pingshan | 102.47 | 25.56 | 1720 | Yunyan87 | Biochar | 1.19 |
| 2019 | Yunnan | Kunming | Luquan | Pingshan | 102.47 | 25.56 | 1720 | Yunyan87 | Biochar | 1.22 |
| 2019 | Yunnan | Kunming | Luquan | Pingshan | 102.47 | 25.56 | 1720 | Yunyan87 | Biochar | 1.29 |
| 2019 | Yunnan | Kunming | Luquan | Pingshan | 102.47 | 25.56 | 1720 | Yunyan87 | Biochar | 1.22 |
| 2019 | Yunnan | Kunming | Luquan | Pingshan | 102.47 | 25.56 | 1720 | Yunyan87 | Biochar | 1.14 |
| 2019 | Yunnan | Kunming | Luquan | Pingshan | 102.47 | 25.56 | 1720 | Yunyan87 | Biochar | 1.35 |
| 2019 | Yunnan | Kunming | Luquan | Pingshan | 102.47 | 25.56 | 1720 | Yunyan87 | Biochar | 1.28 |
| 2019 | Yunnan | Kunming | Luquan | Pingshan | 102.47 | 25.56 | 1720 | Yunyan87 | Biochar | 1.18 |
| 2020 | Yunnan | Kunming | Luquan | Sayingpan | 102.53 | 26.01 | 2250 | Honghuadajinyuan | Biochar | 26.90 |
| 2020 | Yunnan | Kunming | Luquan | Sayingpan | 102.53 | 26.01 | 2250 | Honghuadajinyuan | Biochar | 25.76 |
| 2020 | Yunnan | Kunming | Luquan | Sayingpan | 102.53 | 26.01 | 2250 | Honghuadajinyuan | Biochar | 21.73 |
| 2020 | Yunnan | Kunming | Luquan | Tanglang | 102.29 | 26.19 | 1970 | Honghuadajinyuan | Biochar | 13.71 |
| 2020 | Yunnan | Kunming | Luquan | Tanglang | 102.29 | 26.19 | 1970 | Honghuadajinyuan | Biochar | 16.93 |
| 2020 | Yunnan | Kunming | Luquan | Tanglang | 102.29 | 26.19 | 1970 | Honghuadajinyuan | Biochar | 16.38 |
| 2020 | Yunnan | Kunming | Luquan | Tanglang | 102.29 | 26.19 | 1970 | Yunyan87 | Biochar | 3.84 |
| 2020 | Yunnan | Kunming | Luquan | Tanglang | 102.29 | 26.19 | 1970 | Yunyan87 | Biochar | 4.74 |
| 2020 | Yunnan | Kunming | Luquan | Tanglang | 102.29 | 26.19 | 1970 | Yunyan87 | Biochar | 4.59 |
| 2020 | Yunnan | Kunming | Shilin | Dake | 103.25 | 24.61 | 1643.81 | Honghuadajinyuan | Biochar | 30.36 |
| 2020 | Yunnan | Kunming | Shilin | Dake | 103.25 | 24.61 | 1643.81 | Honghuadajinyuan | Biochar | 26.71 |
| 2020 | Yunnan | Kunming | Shilin | Dake | 103.25 | 24.61 | 1643.81 | Honghuadajinyuan | Biochar | 23.19 |
| 2019 | Yunnan | Kunming | Shilin | Dake | 103.25 | 24.61 | 1643.81 | K326 | Biochar | 1.38 |
| 2019 | Yunnan | Kunming | Shilin | Dake | 103.25 | 24.61 | 1643.81 | K326 | Biochar | 1.41 |
| 2019 | Yunnan | Kunming | Shilin | Dake | 103.25 | 24.61 | 1643.81 | K326 | Biochar | 1.45 |
| 2019 | Yunnan | Kunming | Shilin | Dake | 103.25 | 24.61 | 1643.81 | K326 | Biochar | 1.32 |
| 2019 | Yunnan | Kunming | Shilin | Dake | 103.25 | 24.61 | 1643.81 | K326 | Biochar | 1.37 |
| 2019 | Yunnan | Kunming | Shilin | Dake | 103.25 | 24.61 | 1643.81 | K326 | Biochar | 1.56 |
| 2019 | Yunnan | Kunming | Shilin | Dake | 103.25 | 24.61 | 1643.81 | K326 | Biochar | 1.91 |
| 2019 | Yunnan | Kunming | Shilin | Dake | 103.25 | 24.61 | 1643.81 | K326 | Biochar | 1.97 |
| 2019 | Yunnan | Kunming | Shilin | Dake | 103.25 | 24.61 | 1643.81 | K326 | Biochar | 1.98 |
| 2019 | Yunnan | Kunming | Shilin | Guishan | 103.56 | 24.65 | 1850 | K326 | Biochar | 1.32 |
| 2019 | Yunnan | Kunming | Shilin | Guishan | 103.56 | 24.65 | 1850 | K326 | Biochar | 1.37 |
| 2019 | Yunnan | Kunming | Shilin | Guishan | 103.56 | 24.65 | 1850 | K326 | Biochar | 1.42 |
| 2019 | Yunnan | Kunming | Shilin | Guishan | 103.56 | 24.65 | 1850 | K326 | Biochar | 1.32 |
| 2019 | Yunnan | Kunming | Shilin | Guishan | 103.56 | 24.65 | 1850 | K326 | Biochar | 1.27 |
| 2019 | Yunnan | Kunming | Shilin | Guishan | 103.56 | 24.65 | 1850 | K326 | Biochar | 1.42 |
| 2019 | Yunnan | Kunming | Shilin | Guishan | 103.56 | 24.65 | 1850 | K326 | Biochar | 1.69 |
| 2019 | Yunnan | Kunming | Shilin | Guishan | 103.56 | 24.65 | 1850 | K326 | Biochar | 1.74 |
| 2019 | Yunnan | Kunming | Shilin | Guishan | 103.56 | 24.65 | 1850 | K326 | Biochar | 1.71 |
| 2020 | Yunnan | Kunming | Shilin | Xijiekou | 103.52 | 24.83 | 1997.2 | Honghuadajinyuan | Biochar | 13.20 |
| 2020 | Yunnan | Kunming | Shilin | Xijiekou | 103.52 | 24.83 | 1997.2 | Honghuadajinyuan | Biochar | 16.84 |
| 2020 | Yunnan | Kunming | Shilin | Xijiekou | 103.52 | 24.83 | 1997.2 | Honghuadajinyuan | Biochar | 19.23 |
| 2020 | Yunnan | Kunming | Shilin | Chanhu | 103.41 | 24.71 | 1838.67 | NC102 | Biochar | 4.33 |
| 2020 | Yunnan | Kunming | Shilin | Chanhu | 103.41 | 24.71 | 1838.67 | NC102 | Biochar | 6.21 |
| 2020 | Yunnan | Kunming | Shilin | Chanhu | 103.41 | 24.71 | 1838.67 | NC102 | Biochar | 9.25 |
| 2020 | Yunnan | Kunming | Shilin | Chanhu | 103.41 | 24.71 | 1838.67 | NC102 | Biochar | 6.94 |
| 2020 | Yunnan | Kunming | Shilin | Chanhu | 103.41 | 24.71 | 1838.67 | NC102 | Biochar | 7.26 |
| 2020 | Yunnan | Kunming | Shilin | Chanhu | 103.41 | 24.71 | 1838.67 | NC102 | Biochar | 8.86 |
| 2019 | Yunnan | Kunming | Songming | - | 103.05 | 25.34 | 2100 | K326 | Biochar | 2.41 |
| 2019 | Yunnan | Kunming | Songming | - | 103.05 | 25.34 | 2100 | K326 | Biochar | 2.51 |
| 2019 | Yunnan | Kunming | Songming | - | 103.05 | 25.34 | 2100 | K326 | Biochar | 2.59 |
| 2019 | Yunnan | Kunming | Songming | - | 103.05 | 25.34 | 2100 | K326 | Biochar | 2.52 |
| 2019 | Yunnan | Kunming | Songming | - | 103.05 | 25.34 | 2100 | K326 | Biochar | 2.34 |
| 2019 | Yunnan | Kunming | Songming | - | 103.05 | 25.34 | 2100 | K326 | Biochar | 2.70 |
| 2019 | Yunnan | Kunming | Songming | - | 103.05 | 25.34 | 2100 | K326 | Biochar | 2.80 |
| 2019 | Yunnan | Kunming | Songming | - | 103.05 | 25.34 | 2100 | K326 | Biochar | 2.73 |
| 2019 | Yunnan | Kunming | Songming | - | 103.05 | 25.34 | 2100 | K326 | Biochar | 2.89 |
| 2019 | Yunnan | Kunming | Songming | Niulanjiang | 103.14 | 25.28 | 1850 | K326 | Biochar | 6.27 |
| 2019 | Yunnan | Kunming | Songming | Niulanjiang | 103.14 | 25.28 | 1850 | K326 | Biochar | 6.55 |
| 2019 | Yunnan | Kunming | Songming | Niulanjiang | 103.14 | 25.28 | 1850 | K326 | Biochar | 6.62 |
| 2019 | Yunnan | Kunming | Xundian | - | 103.26 | 25.56 | 1762 | Yunyan87 | Biochar | 3.72 |
| 2019 | Yunnan | Kunming | Xundian | - | 103.26 | 25.56 | 1762 | Yunyan87 | Biochar | 3.69 |
| 2019 | Yunnan | Kunming | Xundian | - | 103.26 | 25.56 | 1762 | Yunyan87 | Biochar | 3.75 |
| 2019 | Yunnan | Kunming | Xundian | - | 103.26 | 25.56 | 1762 | Yunyan87 | Biochar | 3.56 |
| 2019 | Yunnan | Kunming | Xundian | - | 103.26 | 25.56 | 1762 | Yunyan87 | Biochar | 3.47 |
| 2019 | Yunnan | Kunming | Xundian | - | 103.26 | 25.56 | 1762 | Yunyan87 | Biochar | 3.62 |
| 2019 | Yunnan | Kunming | Xundian | - | 103.26 | 25.56 | 1762 | Yunyan87 | Biochar | 4.21 |
| 2019 | Yunnan | Kunming | Xundian | - | 103.26 | 25.56 | 1762 | Yunyan87 | Biochar | 4.36 |
| 2019 | Yunnan | Kunming | Xundian | - | 103.26 | 25.56 | 1762 | Yunyan87 | Biochar | 4.43 |
| 2019 | Yunnan | Kunming | Xundian | Qixing | 103.34 | 25.54 | 1990 | Yunyan87 | Biochar | 2.41 |
| 2019 | Yunnan | Kunming | Xundian | Qixing | 103.34 | 25.54 | 1990 | Yunyan87 | Biochar | 2.11 |
| 2019 | Yunnan | Kunming | Xundian | Qixing | 103.34 | 25.54 | 1990 | Yunyan87 | Biochar | 2.09 |
| 2019 | Yunnan | Kunming | Xundian | Qixing | 103.34 | 25.54 | 1990 | Yunyan87 | Biochar | 2.62 |
| 2019 | Yunnan | Kunming | Xundian | Qixing | 103.34 | 25.54 | 1990 | Yunyan87 | Biochar | 2.54 |
| 2019 | Yunnan | Kunming | Xundian | Qixing | 103.34 | 25.54 | 1990 | Yunyan87 | Biochar | 2.60 |
| 2019 | Yunnan | Kunming | Xundian | Qixing | 103.34 | 25.54 | 1990 | Yunyan87 | Biochar | 2.81 |
| 2019 | Yunnan | Kunming | Xundian | Qixing | 103.34 | 25.54 | 1990 | Yunyan87 | Biochar | 2.73 |
| 2019 | Yunnan | Kunming | Xundian | Qixing | 103.34 | 25.54 | 1990 | Yunyan87 | Biochar | 2.69 |
| 2019 | Yunnan | Kunming | Yiliang | Genjiayin | 103.24 | 25.03 | 1492 | K326 | Biochar | 4.20 |
| 2019 | Yunnan | Kunming | Yiliang | Genjiayin | 103.24 | 25.03 | 1492 | K326 | Biochar | 3.98 |
| 2019 | Yunnan | Kunming | Yiliang | Genjiayin | 103.24 | 25.03 | 1492 | K326 | Biochar | 3.90 |
| 2019 | Yunnan | Kunming | Yiliang | Jiuxiang | 103.39 | 25.10 | 1920 | K326 | Biochar | 1.49 |
| 2019 | Yunnan | Kunming | Yiliang | Jiuxiang | 103.39 | 25.10 | 1920 | K326 | Biochar | 1.13 |
| 2019 | Yunnan | Kunming | Yiliang | Jiuxiang | 103.39 | 25.10 | 1920 | K326 | Biochar | 1.23 |
| 2019 | Yunnan | Kunming | Yiliang | Jiuxiang | 103.39 | 25.10 | 1920 | K326 | Biochar | 1.52 |
| 2019 | Yunnan | Kunming | Yiliang | Jiuxiang | 103.39 | 25.10 | 1920 | K326 | Biochar | 1.47 |
| 2019 | Yunnan | Kunming | Yiliang | Jiuxiang | 103.39 | 25.10 | 1920 | K326 | Biochar | 1.31 |
| 2019 | Yunnan | Kunming | Yiliang | Jiuxiang | 103.39 | 25.10 | 1920 | K326 | Biochar | 2.00 |
| 2019 | Yunnan | Kunming | Yiliang | Jiuxiang | 103.39 | 25.10 | 1920 | K326 | Biochar | 1.93 |
| 2019 | Yunnan | Kunming | Yiliang | Jiuxiang | 103.39 | 25.10 | 1920 | K326 | Biochar | 1.90 |
| 2020 | Yunnan | Kunming | Yiliang | Zhushan | 103.12 | 24.58 | 1650 | Honghuadajinyuan | Biochar | 15.60 |
| 2020 | Yunnan | Kunming | Yiliang | Zhushan | 103.12 | 24.58 | 1650 | Honghuadajinyuan | Biochar | 8.60 |
| 2020 | Yunnan | Kunming | Yiliang | Zhushan | 103.12 | 24.58 | 1650 | Honghuadajinyuan | Biochar | 6.40 |
| 2020 | Yunnan | Kunming | Yiliang | Zhushan | 103.12 | 24.58 | 1650 | Honghuadajinyuan | Biochar | 16.90 |
| 2020 | Yunnan | Kunming | Yiliang | Zhushan | 103.12 | 24.58 | 1650 | Honghuadajinyuan | Biochar | 17.90 |
| 2020 | Yunnan | Kunming | Yiliang | Zhushan | 103.12 | 24.58 | 1650 | Honghuadajinyuan | Biochar | 19.60 |
| 2020 | Yunnan | Kunming | Yiliang | Zhushan | 103.12 | 24.58 | 1650 | Honghuadajinyuan | Biochar | 13.40 |
| 2020 | Yunnan | Kunming | Yiliang | Zhushan | 103.12 | 24.58 | 1650 | Honghuadajinyuan | Biochar | 12.70 |
| 2020 | Yunnan | Kunming | Yiliang | Zhushan | 103.12 | 24.58 | 1650 | Honghuadajinyuan | Biochar | 18.80 |
| 2020 | Yunnan | Kunming | Yiliang | Zhushan | 103.12 | 24.58 | 1650 | Honghuadajinyuan | Biochar | 6.90 |
| 2020 | Yunnan | Kunming | Yiliang | Zhushan | 103.12 | 24.58 | 1650 | Honghuadajinyuan | Biochar | 17.30 |
| 2020 | Yunnan | Kunming | Yiliang | Zhushan | 103.12 | 24.58 | 1650 | Honghuadajinyuan | Biochar | 18.80 |
| 2020 | Yunnan | Kunming | Yiliang | Zhushan | 103.12 | 24.58 | 1650 | Honghuadajinyuan | Biochar | 7.70 |
| 2020 | Yunnan | Kunming | Yiliang | Zhushan | 103.12 | 24.58 | 1650 | Honghuadajinyuan | Biochar | 7.10 |
| 2020 | Yunnan | Kunming | Yiliang | Zhushan | 103.12 | 24.58 | 1650 | Honghuadajinyuan | Biochar | 20.40 |
| 2020 | Yunnan | Kunming | Yiliang | Zhushan | 103.12 | 24.58 | 1650 | Honghuadajinyuan | Biochar | 8.90 |
| 2020 | Yunnan | Kunming | Yiliang | Zhushan | 103.12 | 24.58 | 1650 | Honghuadajinyuan | Biochar | 17.40 |
| 2020 | Yunnan | Kunming | Yiliang | Zhushan | 103.12 | 24.58 | 1650 | Honghuadajinyuan | Biochar | 10.90 |
| 2020 | Yunnan | Kunming | Yiliang | Zhushan | 103.12 | 24.58 | 1650 | Honghuadajinyuan | Biochar | 36.84 |
| 2020 | Yunnan | Kunming | Yiliang | Zhushan | 103.12 | 24.58 | 1650 | Honghuadajinyuan | Biochar | 39.01 |
| 2020 | Yunnan | Kunming | Yiliang | Zhushan | 103.12 | 24.58 | 1650 | Honghuadajinyuan | Biochar | 26.68 |
| 2020 | Yunnan | Kunming | Yiliang | Zhushan | 103.12 | 24.58 | 1650 | Honghuadajinyuan | Biochar | 30.02 |
| 2020 | Yunnan | Kunming | Yiliang | Zhushan | 103.12 | 24.58 | 1650 | Honghuadajinyuan | Biochar | 36.37 |
| 2020 | Yunnan | Kunming | Yiliang | Zhushan | 103.12 | 24.58 | 1650 | Honghuadajinyuan | Biochar | 39.41 |
| 2021 | Yunnan | Kunming | Yiliang | Zhushan | 103.12 | 24.58 | 1650 | Honghuadajinyuan | Biochar | 8.60 |
| 2021 | Yunnan | Kunming | Yiliang | Zhushan | 103.12 | 24.58 | 1650 | Honghuadajinyuan | Biochar | 8.20 |
| 2021 | Yunnan | Kunming | Yiliang | Zhushan | 103.12 | 24.58 | 1650 | Honghuadajinyuan | Biochar | 10.30 |
| 2021 | Yunnan | Kunming | Yiliang | Zhushan | 103.12 | 24.58 | 1650 | Honghuadajinyuan | Biochar | 10.70 |
| 2021 | Yunnan | Kunming | Yiliang | Zhushan | 103.12 | 24.58 | 1650 | Honghuadajinyuan | Biochar | 10.30 |
| 2021 | Yunnan | Kunming | Yiliang | Zhushan | 103.12 | 24.58 | 1650 | Honghuadajinyuan | Biochar | 8.80 |
| 2021 | Yunnan | Kunming | Yiliang | Zhushan | 103.12 | 24.58 | 1650 | Honghuadajinyuan | Biochar | 7.00 |
| 2021 | Yunnan | Kunming | Yiliang | Zhushan | 103.12 | 24.58 | 1650 | Honghuadajinyuan | Biochar | 9.70 |
| 2021 | Yunnan | Kunming | Yiliang | Zhushan | 103.12 | 24.58 | 1650 | Honghuadajinyuan | Biochar | 5.60 |
| 2021 | Yunnan | Kunming | Yiliang | Zhushan | 103.12 | 24.58 | 1650 | Honghuadajinyuan | Biochar | 9.80 |
| 2021 | Yunnan | Kunming | Yiliang | Zhushan | 103.12 | 24.58 | 1650 | Honghuadajinyuan | Biochar | 10.00 |

Note: Sampling sites within the same town are adjacent plots, so the latitude, longitude, and elevation are the same.

**Table S2. Welch's Test Results for Fungal Functions**

| Order | Fungal function | Biochar Mean | Control Mean | Statistic | p.value | Conf.low | Conf.high |
| --- | --- | --- | --- | --- | --- | --- | --- |
| 1 | ANAGLYCOLYSIS-PWY | 12348.48 | 11439.71 | 0.92 | 0.42 | -2184.00 | 4001.54 |
| 2 | CALVIN-PWY | 21487.67 | 20954.05 | 0.14 | 0.90 | -13229.52 | 14296.75 |
| 3 | COA-PWY | 12501.18 | 11457.94 | 0.46 | 0.68 | -7811.51 | 9897.97 |
| 4 | COLANSYN-PWY | 11776.83 | 10121.80 | 1.52 | 0.20 | -1377.05 | 4687.11 |
| 5 | FAO-PWY | 30006.15 | 28213.32 | 0.37 | 0.74 | -16657.24 | 20242.89 |
| 6 | FASYN-ELONG-PWY | 105882.63 | 141481.88 | -0.69 | 0.53 | -190860.68 | 119662.19 |
| 7 | GLUCOSE1PMETAB-PWY | 11047.83 | 10462.30 | 0.21 | 0.85 | -10027.66 | 11198.72 |
| 8 | GLYCOCAT-PWY | 13274.00 | 12525.98 | 0.25 | 0.82 | -10577.47 | 12073.51 |
| 9 | GLYCOGENSYNTH-PWY | 396.70 | 885.68 | -0.69 | 0.56 | -3415.86 | 2437.89 |
| 10 | GLYOXYLATE-BYPASS | 31246.81 | 29327.12 | 0.38 | 0.74 | -17545.83 | 21385.22 |
| 11 | HEME-BIOSYNTHESIS-II | 16607.60 | 15779.27 | 0.32 | 0.77 | -8696.43 | 10353.11 |
| 12 | HSERMETANA-PWY | 1.00 | 8.00 | -1.30 | 0.32 | -28.74 | 14.74 |
| 13 | LEU-DEG2-PWY | 16618.52 | 19801.53 | -0.64 | 0.56 | -17133.58 | 10767.56 |
| 14 | LIPASYN-PWY | 158.68 | 89.69 | 0.72 | 0.52 | -229.72 | 367.70 |
| 15 | NAD-BIOSYNTHESIS-II | 2177.12 | 2390.61 | -0.23 | 0.83 | -3059.04 | 2632.06 |
| 16 | NONOXIPENT-PWY | 24963.66 | 23546.00 | 0.42 | 0.70 | -9358.76 | 12194.07 |
| 17 | P221-PWY | 412.23 | 416.61 | -0.02 | 0.98 | -536.32 | 527.56 |
| 18 | PANTO-PWY | 18955.72 | 18325.22 | 0.22 | 0.84 | -9295.22 | 10556.21 |
| 19 | PANTOSYN-PWY | 13247.65 | 10668.34 | 2.07 | 0.14 | -1665.17 | 6823.78 |
| 20 | PENTOSE-P-PWY | 13405.96 | 12248.62 | 1.00 | 0.39 | -2482.13 | 4796.81 |
| 21 | PHOSLIPSYN-PWY | 168.88 | 51.38 | 1.28 | 0.31 | -222.50 | 457.49 |
| 22 | PWY-3781 | 32268.28 | 29563.98 | 0.62 | 0.59 | -12727.94 | 18136.54 |
| 23 | PWY-4984 | 17631.11 | 17339.82 | 0.09 | 0.93 | -10503.85 | 11086.43 |
| 24 | PWY-5189 | 18358.78 | 17422.69 | 0.32 | 0.77 | -9738.26 | 11610.44 |
| 25 | PWY-5651 | 12937.63 | 12554.74 | 0.11 | 0.92 | -12598.44 | 13364.22 |
| 26 | PWY-5659 | 20276.96 | 19460.23 | 0.27 | 0.81 | -10821.38 | 12454.85 |
| 27 | PWY-5667 | 8882.25 | 7983.14 | 0.91 | 0.44 | -2442.43 | 4240.65 |
| 28 | PWY-5695 | 366.36 | 1003.84 | -0.82 | 0.50 | -3893.86 | 2618.90 |
| 29 | PWY-5705 | 31.91 | 17.98 | 0.72 | 0.52 | -46.34 | 74.20 |
| 30 | PWY-5920 | 17438.75 | 16559.79 | 0.32 | 0.77 | -9187.67 | 10945.60 |
| 31 | PWY-5989 | 2682.10 | 2758.79 | -0.07 | 0.95 | -4282.71 | 4129.33 |
| 32 | PWY-6125 | 19583.89 | 18692.83 | 0.31 | 0.78 | -8721.27 | 10503.40 |
| 33 | PWY-6126 | 23227.98 | 22415.36 | 0.23 | 0.83 | -10817.85 | 12443.09 |
| 34 | PWY-621 | 18337.85 | 17322.91 | 0.23 | 0.84 | -15447.65 | 17477.53 |
| 35 | PWY-6317 | 16043.40 | 15544.41 | 0.15 | 0.90 | -12675.90 | 13673.86 |
| 36 | PWY-6545 | 3510.40 | 3641.46 | -0.09 | 0.93 | -5207.97 | 4945.85 |
| 37 | PWY-6608 | 367.79 | 1024.30 | -0.83 | 0.49 | -3997.34 | 2684.32 |
| 38 | PWY-6609 | 246.26 | 704.85 | -0.83 | 0.49 | -2778.31 | 1861.14 |
| 39 | PWY-6737 | 13039.47 | 12671.58 | 0.10 | 0.93 | -13365.35 | 14101.13 |
| 40 | PWY-7007 | 19369.73 | 18335.81 | 0.36 | 0.75 | -9473.19 | 11541.04 |
| 41 | PWY-7111 | 24475.53 | 23184.80 | 0.34 | 0.76 | -12964.81 | 15546.28 |
| 42 | PWY-7184 | 20488.98 | 19237.52 | 0.47 | 0.68 | -7988.84 | 10491.75 |
| 43 | PWY-7196 | 1.17 | 9.33 | -1.30 | 0.32 | -33.51 | 17.19 |
| 44 | PWY-7197 | 17222.78 | 16151.55 | 0.48 | 0.67 | -6652.71 | 8795.18 |
| 45 | PWY-7198 | 2802.88 | 2893.34 | -0.08 | 0.94 | -4240.85 | 4059.93 |
| 46 | PWY-7208 | 18524.70 | 17409.47 | 0.44 | 0.70 | -8045.58 | 10276.04 |
| 47 | PWY-7209 | 339.66 | 370.63 | -0.15 | 0.89 | -718.15 | 656.21 |
| 48 | PWY-7210 | 3737.00 | 3895.65 | -0.11 | 0.92 | -5384.70 | 5067.41 |
| 49 | PWY-7219 | 28396.73 | 29988.97 | -0.26 | 0.81 | -20789.50 | 17605.03 |
| 50 | PWY-7220 | 23544.80 | 21990.06 | 0.57 | 0.61 | -7049.48 | 10158.95 |
| 51 | PWY-7221 | 18774.92 | 18131.95 | 0.22 | 0.84 | -9272.93 | 10558.87 |
| 52 | PWY-7222 | 23544.80 | 21990.06 | 0.57 | 0.61 | -7049.48 | 10158.95 |
| 53 | PWY-7228 | 20623.66 | 19723.45 | 0.30 | 0.78 | -8939.33 | 10739.75 |
| 54 | PWY-7229 | 23831.25 | 23196.24 | 0.17 | 0.88 | -12257.84 | 13527.86 |
| 55 | PWY-7323 | 10393.03 | 9159.70 | 0.96 | 0.40 | -2418.27 | 4884.92 |
| 56 | PWY-7328 | 8834.08 | 11146.49 | -0.60 | 0.58 | -12986.07 | 8361.26 |
| 57 | PWY-7347 | 4.50 | 1.50 | 0.65 | 0.58 | -15.47 | 21.46 |
| 58 | PWY-7385 | 10621.03 | 9537.41 | 1.47 | 0.24 | -1233.18 | 3400.40 |
| 59 | PWY-7431 | 34323.54 | 32626.67 | 0.17 | 0.88 | -38062.97 | 41456.72 |
| 60 | PWY-7663 | 1871.93 | 1924.47 | -0.06 | 0.95 | -3055.60 | 2950.54 |
| 61 | PWY-922 | 15932.49 | 15059.43 | 0.36 | 0.75 | -8209.88 | 9956.00 |
| 62 | PWY0-1319 | 8882.25 | 7983.14 | 0.91 | 0.44 | -2442.43 | 4240.65 |
| 63 | PWY0-166 | 19536.75 | 18358.42 | 0.45 | 0.69 | -8041.87 | 10398.54 |
| 64 | PWY4FS-7 | 102.04 | 30.90 | 1.28 | 0.31 | -134.99 | 277.26 |
| 65 | PWY4FS-8 | 102.04 | 30.90 | 1.28 | 0.31 | -134.99 | 277.26 |
| 66 | SER-GLYSYN-PWY | 22078.64 | 21447.33 | 0.17 | 0.88 | -13060.52 | 14323.13 |
| 67 | SO4ASSIM-PWY | 16426.05 | 15570.95 | 0.32 | 0.77 | -8769.46 | 10479.66 |
| 68 | SUCSYN-PWY | 14.97 | 5.00 | 0.65 | 0.58 | -51.46 | 71.40 |
| 69 | TCA | 940.55 | 1375.35 | -0.47 | 0.68 | -3913.63 | 3044.03 |
| 70 | THRESYN-PWY | 17971.71 | 17593.50 | 0.12 | 0.91 | -10055.14 | 10811.57 |
| 71 | TRNA-CHARGING-PWY | 23934.50 | 23177.07 | 0.18 | 0.87 | -14075.14 | 15589.99 |
| 72 | TYRFUMCAT-PWY | 18806.67 | 22751.40 | -0.51 | 0.65 | -29965.59 | 22076.13 |
| 73 | VALSYN-PWY | 22370.70 | 21411.86 | 0.26 | 0.81 | -12404.87 | 14322.55 |

Note: In the table, Biochar Mean represents the average abundance of fungal functions associated with the biochar treatment, and Control Mean represents the average abundance of fungal functions associated with the control treatment. Statistic indicates the t-value from the t-test, p.value represents the two-tailed p-value corresponding to the t-value, and Parameter represents the degrees of freedom. Conf.low and Conf.high indicate the lower and upper limits of the estimated confidence interval, respectively.
